# Metacognitive monitoring and metacognitive strategies of gifted and average children on dealing with deductive reasoning task

**DOI:** 10.16910/jemr.14.4.1

**Published:** 2021-09-14

**Authors:** Ondřeji Straka, Šárka Portešová, Daniela Halámková, Michal Jabůrek

**Affiliations:** Masaryk University, Brno, Czechia

**Keywords:** individual differences, metacognition, gifted children, deductive reasoning, mental models, eye tracking, gaze transition entropy, reading

## Abstract

In this paper, we inquire into possible differences between children with exceptionally high intellectual abilities and their average peers as regards metacognitive monitoring and related metacognitive strategies. The question whether gifted children surpass their typically developing peers not only in the intellectual abilities, but also in their level of metacognitive skills, has not been convincingly answered so far. We sought to examine the indicators of metacognitive behavior by means of eye-tracking technology and to compare these findings with the participants’ subjective confidence ratings. Eye-movement data of gifted and average students attending final grades of primary school (4th and 5th grades) were recorded while they dealt with a deductive reasoning task, and four metrics supposed to bear on metacognitive skills, namely the overall trial duration, mean fixation duration, number of regressions and normalized gaze transition entropy, were analyzed. No significant differences between gifted and average children were found in the normalized gaze transition entropy, in mean fixation duration, nor - after controlling for the trial duration – in number of regressions. Both groups of children differed in the time devoted to solving the task. Both groups significantly differed in the association between time devoted to the task and the participants’ subjective confidence rating, where only the gifted children tended to devote more time when they felt less confident. Several implications of these findings are discussed.

## Introduction

The term metacognition, denoting the ability to reflect upon cognitive phenomena (both one’s own and those of other people) and to adjust one’s learning and problem solving according to this knowledge, is at present firmly established within the fields of educational science (25 ), psychology (9), as well as neuroscience (19; 47). In addition to the theoretical importance of the construct, metacognition has also a tremendous practical impact since it has been repeatedly demonstrated that this competence can be developed and fostered on purpose by means of special programs integrated in standard or special education and that such interventions significantly increase the overall level of students’ scholastic achievement (30; 43 ; 56; 72).

According to a widely used taxonomy, metacognitive phenomena are usually classified into two broad classes (3; 73; 78). *Metacognitive knowledge* (also known as declarative forms of metacognition) denotes the person’s information and believes about cognitive activities, both his/her own and those pertaining to human mind in general (3; 14). An example of the metacognitive knowledge bearing on the subject’s own mind may be the awareness of the student that his memory capacity is lower (or higher) in comparison with typical schoolmates, or a belief of another student that she can memorize verbal material more easily than pictorial one.

Procedural forms of metacognition – often termed as *metacognitive skills* – comprise in the first place the ability to actually use various strategies that take into account the strengths and limitations of one’s own cognition in order to improve the process of learning and problem solving (28 ). For instance, when preparing for an exam, students may not read the text indiscriminately but monitor for its difficulty and adapt their reading accordingly (e.g. by skipping easier or already known parts, and contrariwise reading the difficult parts more slowly and more attentively etc.). The second important facet of procedural metacognition is metacognitive monitoring (62 ). This term denotes a set of mental processes, comprising the initial appraisal of a cognitive task in terms of its difficulty, continuous checking of the progress during the task execution, detecting the presence of obstacles that may hamper the attainment of the learning or problem-solving goal. It is obvious that both facets of procedural metacognition are tightly intertwined, because the information gained as a result of metacognitive monitoring constitutes the base for choosing optimal strategy, and each strategy, once chosen, is conversely subject to continuous monitoring.

### Metacognition and intellectual giftedness

One of the crucial issues in the study of metacognition has been its relation to the level of cognitive abilities and their differences among individuals. In other words, the question stands whether the people with high intellectual abilities show also a high level of metacognition and vice versa. Several competing models have been proposed to tackle this issue, and there exists some occasional evidence both for the notion that metacognitive abilities are essentially just one facet of general intelligence (18) and for the approach that sees intelligence and metacognition as two separate abilities, completely independent of one another (5). However, on the strength of many studies summed up by Veenman (71), it seems that the relation between intelligence and metacognition is best approached by so called *mixed model*. This model concedes that intelligence and metacognition share some amount of common variance and it assumes that these faculties are somehow interrelated (though it is not a “theory” in terms of providing some neurological or other causal explanation for this fact). The strength of this connection is, however, relatively week, and this fact manifests itself by way of low or medium correlations between the measures of both faculties (28; 71).

The consequences ensuing from the mixed model are particularly important to a specific population of intellectually gifted children, as it presumes that even within this population, there is some variation in the level of metacognitive abilities. Therefore, one may expect to find some intellectually gifted children with low level of metacognition, although such condition should be significantly less common as compared to the combination of both faculties in the above-average level. This assumption dovetails with the fact, that a certain number of gifted children (whose abilities were corroborated by testing) actually go through various learning problems and their school results are significantly worse than what could be expected given the level of their intelligence. This condition is usually termed underachievement in the literature on gifted education (65), and although it may be caused by a host of other factors, the insufficient level of metacognitive skills has been proposed as a possible explanation (74).

The question of how much gifted children differ from their average peers with regard to their metacognitive abilities and whether their metacognition is specific in some respects (e.g. whether they tend to systematically surpass the general population in some specific facets of metacognition, while acting on the same level in others) has been intensely studied in the past (2; 4 ; 24; 67). This research has rather convincingly demonstrated that gifted children tend to surpass their peers in the domain of metacognitive knowledge. As to the metacognitive skills, the results were obviously less conclusive, with most (though by no means all) studies reporting no significant differences between both groups.

However, we believe that the absence of essential differences between gifted and average children is not so indisputable and that the topic merits further investigation, especially in view of the advances in the methodology of research on metacognition which have been achieved in the recent years. The reason why studies carried out in the past might have missed some actually existing effects of giftedness on the metacognitive skills is that a great deal of them were problematic in two aspects:

First, as Prins et al. (53) pointed out, experiments on metacognition are principally operative only with sufficiently difficult materials used to stimulate learning or problem solving. When the presented tasks are too easy, they do not call for any sophisticated strategy and participants can usually solve them automatically and with no need to employ any metacognitive activity. This might have been precisely the case of many metacognitive studies carried out in the past, which excessively used simple memorizing tasks as their principal stimuli and often required no deeper thinking or reasoning activity from their participants (46; 63).

Second, in a substantial part of extant metacognitive research, the data on the participants’ metacognition have been obtained primarily through various questionnaires, (semi)structured interviews and similar, so called off-line methods (i.e. methods, that are applied before, or – more frequently – after the stimulus task itself, with a considerable delay). In the course of last two decades, it has been repeatedly demonstrated that this class of methods in general suffer from both low reliability and validity (75; 77; 81). A partial exception is represented by so called local calibration measures, which are obtained by asking participants to give a simple subjective difficulty rating of each task in some broader set *immediately after completing that task* (46; 67; for concrete example, see Method section of this paper). In view of these problems, it is generally advisable to use on-line methods – i.e. methods that register metacognitive data directly in the course of processing the stimulus tasks – whenever it is possible. There are several well-proven methods of this sort, such as analysis of think-aloud protocols, analysis of computer log-files and others (for a detailed description of these techniques see 75). Eye-tracking also positively falls into this category. Thus, it seems a paradox that, as van Gog and Jarodzka (70) pointed out, this method has been employed much more frequently to study the cognition itself, in contrast with its comparatively rare application in the research on metacognition.

These unresolved issues inspired our own research. In this paper, we are seeking to re-examine the question of whether gifted children differ from their average counterparts in the level of metacognitive skills. To obviate the pitfalls mentioned above, we deliberately chose rather difficult stimulus tasks calling for complicated relational reasoning. We opted for eye-tracking as a primary source of data and we sought to compare these with the results of the participants’ subjective difficulty ratings.

### Mental models theory and relational reasoning

When choosing a set of reasoning tasks to elicit metacognitive behavior, and aiming at the tasks to be suitable for this purpose as to their character and level of difficulty, one can hardly do without a sound theoretical footing. One of the approaches which seek to explain what is going on in the human mind during the process of logical reasoning is the mental model theory (in short, “model theory”), put forth by Johnson-Laird and his colleagues (33; 34; 23).

Johnson-Laird (33) posits that people construct in their minds internal representations, which capture objects and entities in the surrounding world and mutual relations among them. These representations are denoted as mental models if they feature two key properties. First, mental models are *iconic* (in the sense Charles Peirce coined this term) – that is, the structure of the model exactly corresponds to the structure of what the model represents. Second, each mental model represents what is common to a distinct set of *possibilities*. Mental models arise as a result of perception, spoken or written description or of a preceding thinking process. According to the view espoused by the model theory, the appraisal of mental models (as to their veracity or falsity) and deliberate conscious manipulation with them or with parts of them constitute the essence of human reasoning.

We may provide, as a concrete example, a short description of a room with some furniture, which gives rise to a simple mental model of the scene. The description consists of several premises:

1. The sofa is to the right of the door.

2. The piano is to the right of the sofa.

3. The filing cabinet is to the left of the door.

Owing to the iconicity of mental models, a number of veracious conclusions that were stated in none of the premises (e.g. The piano is to the right from the door) can be derived. It is, however, important to point out that the mental model is not the same as a visual image. Given the description above, one can conceive a huge, potentially even infinite number of images that are consistent with it. For instance, the sofa may be blue, or it may be red or green; the door and the filling cabinet may be one meter apart, but the distance between them may also be one and a half meter or two meters etc. All these differences combine into a tremendous number of possibilities. However, as we have mentioned, the model theory presumes that the human mind tends to abstract from such superficial qualities and merge all these possibilities into a single model that can subsequently be appraised and/or manipulated (32).

Some descriptions can be unambiguously represented by a single mental model (such as the example in the previous paragraph). In other cases, however, the set of premises is consistent with two or even more equally legitimate models, all of which need to be taken into account in the process of drawing conclusions from the given premises. The following description is a good case in point:

1. The circle is to the left of the cross.

2. The diamond is to the left of the cross.

3. The square is in front of the circle.

4. The triangle is in front of the cross.

What is the relation between the triangle and the square?

**Figure 1.**
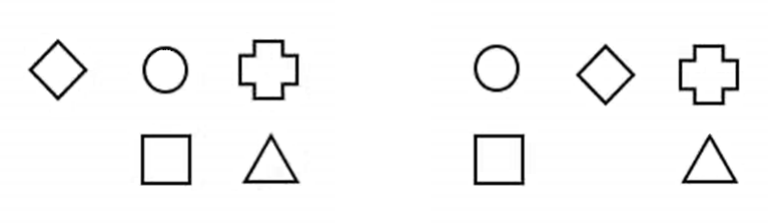
An outline of two mental models congruent with a single set of premises.

This set of premises is congruent with two models, depicted in Figure 1 . Although both models lead to the same answer to the question at hand (the square is to the left from the triangle), the theory predicts that this kind of tasks should be more difficult in comparison with tasks where the description is congruent with only one model. This increase in difficulty is brought about by the necessity to keep both mental models simultaneously in the working memory during the process of making the inferences, thereby significantly taxing one’s cognitive resources. As Johnson-Laird (32) put it, the limited capacity of working memory is the “bottleneck of intelligence” (p. 40). With tasks comprising more complex descriptions, which require a construction and simultaneous manipulation of three or more mental models, the difficulty naturally increases accordingly. This assumption has actually been borne out by research findings, at least in the case of adult participants. When presented with the tasks generating two competing mental models (as in the example illustrated in Figure 1), and the tasks based on a very similar description, in terms of the number of premises and complexity of the scene being described, but requiring only a single mental model, participants are generally faster and achieve a higher percentage of correct answers in the latter case (32; 57 ; 68). For this reason, model theory is very convenient in that it makes it possible to estimate a priori the difficulty of various cognitive tasks.

Given our brief and necessarily somewhat simplifying account of mental model theory, the whole approach may seem too peculiar and too narrow in scope, as one can definitely conceive a plethora of cognitive problems that are not limited to the mutual relations and overall spatial layout of several objects. However, more detailed and more thorough expositions on the subject show that this approach – apart from its application on relational reasoning – has also been successful in explaining many other cognitive phenomena, such as syllogistic and conditional reasoning, using abductions and quantifiers on thinking, refuting false conclusions through counterexamples etc. (8 ; 20; 26; 32; 39). It has been also suggested, that the ability to deal with spatial arrangements in one’s mind is very helpful for all kinds of reasoning that involves transitive relations (Pinker, 49; in logical calculus, the transitivity denotes the property that if A is related to B and B is related to C, than A is related to C; Schmidt, 58). It is also worth noting that besides model theory, there are other influential approaches dealing with human intelligence, which put down the reasoning based on the descriptions of spatial arrays as an indicator of a broader and fundamental cognitive ability. The PASS theory of cognitive processes (17; 21; 45) is a good case in point.

In view of the focus of this study on primary school children, it is also necessary to mention the developmental aspects of mental model theory. Although a bulk of research within this paradigm has been carried out with adult participants, some important studies dealing with pre-adolescent or even pre-school children also exist (12 ; 37; 39). Their findings are all the more important with regard to the long known fact that the capacity of children’s working memory is markedly limited in comparison with adults and it only gradually increases during the years of school attendance (59). One may thus expect that young children will be unable to perform certain operations of logical reasoning at all – especially those that call for the construction of more than a single mental model. Some authors unsurprisingly demonstrated that young children tend to fail systematically in tasks requiring specific trains of mental operations (see 38, for in-depth review). However, it has also been shown that the achievement of very young children can be significantly improved if the assignment avoids using too abstruse concepts or a difficult language and on the other hand, contains premises well grounded in children’s everyday experience (e.g. assignments on conditional reasoning contain items such as “if an animal has stripes, then it is a zebra” and not “if a shape is beige, then it is a pyramid”). What is even more important, Markovits (37) has shown that children as young as 6 years are able – if the assignment is sufficiently concrete – to construct and manipulate at least two distinct mental models simultaneously.

### Hypotheses

Based on the theoretical background presented above, we formulated four specific hypotheses. The measures used for the operationalization are further described in the Method section.

H1: Gifted children will, on account of their globally higher metacognitive skills, correctly monitor for the increased demands presented in the deductive reasoning tasks. This will manifest itself in the higher mean fixation duration, in comparison with average children.

H2: Gifted children will, on account of their globally higher metacognitive skills, correctly identify the necessity to read the task assignments more attentively (as compared to ordinary texts) and therefore will show a higher number of regressions in comparison with average children.

H3: Gifted children will be more systematic in their building of mental models corresponding to the task assignments, which will lead to a lower level of their gaze transition entropy in comparison with average children.

H4: Gifted children will more frequently adjust their processing strategies to the results of their concurrent metacognitive monitoring. Therefore, they will show higher correlation between their ease-of-solution judgements (EoSJ) and time devoted to the processing of the task in comparison with average children.

## Method

### Participants

Initially, 73 students attending the last two grades of primary school (i.e. 4^th^ or 5^th^ grade) in the South Moravian region of the Czech Republic were administered the intelligence test. With respect to the aims of our study, the schools asked to take part in the study were not chosen at random. We deliberately addressed three schools that either provide special classes for gifted education or that have recently participated in projects focused on fostering gifted education in ordinary classrooms (particular schools were attended by 35, 21 and 17 participants, respectively). On these grounds, we could reasonably expect to find classrooms with markedly higher proportion of gifted children as compared to general population. Since the purpose of the research was to compare gifted children with their average peers, we purposely avoided involving students with severe learning problems or with previously diagnosed intellectual disability into this initial phase.

Based on the results of the intelligence test, two groups of participants were established. The group of gifted students consisted of 27 children (17 boys, 10 girls), the control group of average peers comprised 29 children (14 boys, 15 girls). The mean age of the whole sample was 10.8 years, SD = 0.4 years. To all of these children, experimental tasks were presented and the eye movements of these children were recorded. Two participants in the control group did not finish the experiment properly and their data were excluded altogether from any further analyses (in one case, another person disturbed the experiment by entering the room, in second case a participant made an unexpected movement during which he hit the monitor and thus compromised the quality of the record.) After the preprocessing of the records, participants with the tracking ratio less than 85 percent were excluded from the analysis of their eye movement data. Furthermore, we carried out a qualitative inspection of all records aimed at detecting the participants whose records were systematically shifted and/or slanted as a consequence of their changing the body position considerably during the experiment. We identified five participants with records compromised in this way. However, since all these participants had also unacceptable tracking ratio, their identification had no further practical consequence.

We thus distinguish two parts of our sample. The *extended group* consisted of all children that took part in the experiment and finished it properly, regardless of the quality of their eye-tracking record. In the case of these participants, their temporal data (i.e. the length of processing individual trials), their success rate in the tasks comprising the trials and their subjective appraisal of the ease of learning were analyzed. The *basic group* comprised only the subset of participating children with satisfactory quality of their eye-tracking record. For these participants, in addition to the processing of data just mentioned, analyses of several eye-movement related metrics were performed. As far as the narrower part of the sample is concerned, 31 children made up the basic group (16 of them gifted, 15 average controls), with the mean age and standard deviation practically the same as that of the extended sample (the mean age 10.8 yrs., SD = 0.4 yrs.).

### Measure of intellectual ability

To establish the gifted status, the intellectual abilities of children were assessed by means of the Czech version of Cattell’s Fluid Intelligence Test (15). This test is a revised and newly standardized version of the original Culture Fair Intelligence test, authored by Raymond Cattell. As such, the method seeks to measure the abilities of logical reasoning in the purest possible form and to eliminate the influence of different cultural backgrounds or of disparate levels of vocabulary and language faculty among tested individuals. The Czech adaptation was standardized on the sample of 1779 children in the years 2011 – 2013. The manual indicates the reliability (in terms of internal consistency) of the method 0.88.

The question of determining whether a child should or should not be considered gifted is a complicated issue, which has been given a great deal of attention in the literature for many decades (44 ; 48). It is generally acknowledged now that to identify children just on the strength of one IQ value is too narrowing an approach and in real educational setting and/or in psychological counseling it is advisable to take into account multiple information, such as the profile of specific abilities of an individual child, interests and motivation of the child, his or her creativity etc. To meet these ends, it is however necessary to assess children individually via a rather long procedure, which was unfeasible in our research project. Thus, we abided by the traditional approach, which considers children as gifted if their level of IQ is two standard deviations above the average, or higher (42).

Thus, for assigning the participants into the gifted or control (average) group, we set up the following criteria: a) The participants were designated as gifted, if they attained an IQ score equal to 130 or higher, or – in some cases – if they attained the highest possible score for their age cohort. b) The participants with the IQ score between 90 and 110 were assigned to the control (average group). If we used single cut point, individuals on the opposite side of that cut point, which are in fact very similar in their abilities, would be treated as different by way of their assignment to the contrasting groups (6 ). The 20 point gap between the gifted and average group was established arbitrarily to prevent this unwelcome effect. Participants who did not meet the criteria for the inclusion in either group did not take part in the following phases of the study.

### Experimental stimuli

**Figure 2.**
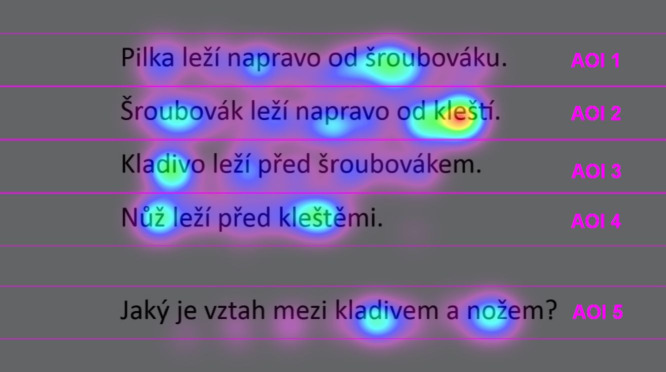
The layout of the screen during the presentation of an experimental trial, with overlaid AOIs and heatmap of the participant #30. Warmer colors indicate greater attention paid to the underlying area on the screen, as derived from the number and duration of gaze fixations. The translation of the utterances used in this example is as follows: 1. The handsaw lies to the right of the screwdriver. 2. The screwdriver lies to the right of the pliers. 3. The hammer lies in front of the screwdriver. 4. The knife lies in front of the pliers. 5. What is the relation between the hammer and the knife?

The stimulus materials comprised separate trials, presented step-by-step to the participants on the computer screen, while their eye-movements were recorded. Each trial had a form of four utterances describing the spatial layout of five different objects in terms of their mutual positions. The descriptions closely mimicked tasks frequently used in the extant mental model literature, particularly we drew on the publications of Johnson-Laird and Byrne (34) and Johnson-Laird (32). Each utterance was presented on a separate line, the lines were set up sufficiently apart to allow a later setting of areas of interest (AOI) for each individual utterance and forestall (undue) blending of gaze data pertaining to these AOIs. On the last, fifth line, a question was displayed, asking the participant about the relation between two particular objects. This relation had never been explicitly stated in any of the preceding utterances, but could be deduced by means of the construction of an appropriate mental model in the mind of the participant. Figure 2 depicts Trial III with an overlaid AOIs and a heat map of a single participant.

Half of the trials contained descriptions calling for building a single mental model (type: A is to the right of B, B is to the right of C, D is in front of A, E is in front of C. What is the relation between D and E?). The other half of the trials comprised descriptions compatible with two different mental models, both of which lead nevertheless to the same answer to a subsequently posed question (type: A is to the right of B, A is to the right of C, D is in front of C, E is in front of E. What is the relation between D and E?).

In general, the contents of the experimental trials were designed in a way that was supposed to eliminate most of the potential confounding factors arising from the differences between intellectual abilities, reading proficiency or prior knowledge. For instance, if the problems we used were so difficult that they would be beyond the capacity of most of the average children to solve them, this fact would inevitably manifest in the nature of the eye movements of these children. Indeed, Hayes et al. (27) reported the effect of varying cognitive abilities on eye-movements during the process of solving intelligence test itself. For this reason, we opted for tasks which are difficult by virtue of their demands on working memory and on the necessity to strategically handle one’s cognitive resources during their solving, yet which are possible to solve for both gifted and average children. We suppose that average children will attain lower number of correct answers overall, but for a typical child from the average group, each individual trial should be possible to solve. In other words, in accordance with the mixed model, the advantage of gifted children on solving the deductive problems of this kind should result from their better use of metacognitive abilities rather than from the employment of some specific neural circuitry which their average peers do not possess at this age.

At the same time, the experimental trials were created with the intention to be relatively insensitive to various factors pertaining to the text properties and known to influence the eye-tracking metrics used in our research. These factors comprise, for instance, word frequency, word predictability, number of meanings, phonological complexity and several others (54). To limit the effects of these factors, the utterances were on purpose designed to contain only frequent, unambiguous, concrete words, without an undue level of phonological complexity. All objects mentioned within one trial were chosen to belong to a common semantic category (e.g. fruits, tools, pieces of clothes etc.). As the utterances had the form of a simple affirmative declarative sentence (or simple question), no effects relating to parsing difficulties should show up either.

To reduce the effect of other potential confounding factors, the order of utterances was counterbalanced. Participants designated with odd numbers were presented the trials from I. through VIII., while participants with event numbers were presented the trials from VIII. through I.

### Apparatus

The experimental trials were displayed on a standard Philips LCD Monitor, set approximately 60 cm in front of the participant. The resolution of the monitor was 1680x1050 pixels, diagonal screen size 22 inches and refresh rate 60 Hz. Participants’ eye movements were recorded through the SMI RED250 eye tracker, with sampling rate set at 250 Hz. Recorded files were transformed by means of the HypOgama application (51) to the format that enabled their import into OGAMA open source software (79). All subsequent analyses of eye-movement data were then realized in this software.

### Measure of success rate

The participants’ answers were recorded and subsequently evaluated, each correctly answered question scored one point. Since the whole procedure comprised eight experimental trials and each the task in the trial had just one correct answer, the participants could attain from 0 to 8 points.

### Measure of subjective metacognitive monitoring

In metacognitive research with older participants (adolescents and adults), a metric called the calibration of performance is often used for the measurement of local, item-by-item metacognitive monitoring. This metric is constructed as follows: the participants give an answer to a particular item (for example a question in a knowledge test, a mathematical problem etc.) and subsequently they estimate the probability of the correctness of their answer. Since the very concept of probability might be largely unfamiliar to primary grade students, especially in the control group, we used a similar, perhaps less known metric, called ease of solution judgement (EoSJ; 61). Immediately after completing each trial, the participants were invited to rate on a scale ranging from 1 to 10 how difficult they perceived this task (1 = most easy; 10 = most difficult). The participants responded verbally, the ratings were then noted down by the examiner.

### On-line measures bearing on metacognitive behavior

As we have already mentioned, eye-tracking has been used rather infrequently in the research on metacognition, as compared to its application to study more general cognitive phenomena (1 ; 70). As a result, the theoretical foundations for choosing appropriate metrics remain still undeveloped. For instance, in a comprehensive handbook by Holmqvist et al. (31), about 120 specific metrics which could be used in eye-tracking research are described. However, in none of these the suitability for metacognitive research is explicitly discussed. To our knowledge, presently there exists no other thorough methodological guideline of this kind.

Consequently, in choosing the measures to register and analyze in our study, we had to rely on our own assumptions about the nature of the experimental tasks and the way they should be solved. We could also draw on the methodologies reported in some of the previous metacognitive studies, so far as their experimental materials bore some resemblance to our own stimuli.

We assume that the main difference between children with high and low level of metacognition consist in the fact that children with low metacognition will construe the experimental problems as simple reading tasks. As was already mentioned, the presented utterances are very undemanding in terms of their phonological, semantic or syntactic complexity and the reading process itself should not call for any sophisticated strategy. Contrarywise, the children with high metacognitive skills should be constantly aware of the additional demands ensuing from the underlying deductive task, they will recognize the necessity to take in each particular piece of information more attentively and to constantly check for what they know and where they may still err. This should show up in the eye-movement measures that proved to be sensitive to the problem complexity and/or ambiguity, not only in metacognitive research, but also in other studies focusing on purely cognitive phenomena. Since, according to the mixed model, intellectually gifted children are expected to show moderately higher level of metacognitive skills, these effects should be apparent also in comparing the group of gifted and average children.

Previous studies dealing with metacognition reported three general types of measures that might be beneficial also in the context of our research: the mean fixation duration (55), the incidence of regressions (35, 36) and some kind of metric capturing the transitions between different parts of stimulus field, reflecting systematic strategic processing of information (41, 40). How these metrics were computed and used in our study is described further below. Besides these measures, we registered and analyzed also overall trial durations, primarily for the sake of testing the hypothesis H4. The rationale of the underlying supposition should be obvious: children with optimal level of metacognitive skills should devote more time to the problems which they perceive as difficult ones, as compared to the problems construed as easy ones.

For the analyses of eye-movements related metrics (i.e. fixation duration, number of regressions and gaze transition entropy), the stimulus field of each trial was divided into five separate areas of interest (AOI). Each of four description utterances made up one AOI, the fifth AOI comprised the final sentence which expressed the problem question. Corresponding AOIs in all eight trials had the same dimensions (in pixels) along the x and y axes and consequently the same area. The division of the stimulus field was carried out in keeping with the recommendations made by Hessels et al. (29), who advised to create the AOIs as big as possible for the sparse stimuli. Minor parts of the screen that did not belong to any AOI (specifically, the strips adjacent to the upper and lower edge of the monitor and the area between the last utterance and the question) were considered blank spaces and were ignored in the derivation of eye-movement measures and in all subsequent analyses. The arrangement of AOIs is shown in Figure 2.

**Time of solution (overall trial duration)**. As no time limit was imposed on the experimental trials and the participants fully controlled the pace of their presentation (see section Procedure for further details), the time each participant devoted to an individual trial as well as the sum of these times across all trials differed markedly. Since for individuals with advanced metacognitive skills it is reasonable to answer the problem question and to proceed to the following trial only after they were sufficiently confident about having arrived at the correct answer, this metric is supposed to be positively indicative of metacognitive behavior. We opted for analyzing the overall time of the trial presentation rather than dwell-time primarily because this metric was available for all participants of the extended sample, including those with insufficient tracking ratio.

**Mean fixation duration**. The mean fixation duration is a measure obtained by averaging the times of individual fixations in one trial and/or in the whole experiment. This metric does not depend on the overall trial duration, since two persons who have spent exactly the same time on a trial may differ markedly in their mean fixation duration. This situation occurs if one person registers high number of relatively short fixations, while the other conversely registers a small number of fixations, which are on average relatively longer. According to extant eye tracking literature, fixation durations tend to be longer if the task at hand is rather complex, ambiguous or demanding, or if the participants perceive it as such (31). On this account, the measure has been suggested and used as an indicator of effective metacognitive monitoring (55). Another reason why metacognitively skillful children should exhibit higher fixation duration comes from the findings of numerous eye-tracking research projects, which were summed up and presented by Rayner (54): it seems that - as compared to simple linear reading - people tend to show longer fixations in tasks which require active searching for information from a wider area. Consequently, children who will monitor for each intermediary step in their building of mental models and based on this monitoring will intentionally search for the information they are uncertain of, are likely to exhibit a higher fixation durations.

Data from all AOIs of each trial were processed together to derive a mean fixation duration of a participant in that trial, subsequently we calculated the grand mean across all eight trials for each participant. To detect fixations, I-DT (Dispersion Threshold Identification) algorithm was used. The parameters of the algorithm were set according to the recommendations given by Popelka (50) at the following values: maximum distance at 15px, minimum number of samples at 20, no merge.

**Number of regressions**. The number of regressions (backward saccades in the opposite direction to the left-right course of reading, aimed at re-fixation of the parts of text already read) has been repeatedly studied in relation to certain cognitive impairments. However, some previous studies showed that in healthy population the increased number of regression signals more deliberate and attentive reading (60). Regressions can be analyzed on various levels – within words, between words within a sentence, or – in the case of stimuli comprising longer texts – even within whole paragraphs. For our purposes, regressions between words within one sentence (comprising a single AOI) were considered most relevant. This type of regressions reflects sentence integration processes, i.e. understanding how individual words in a sentence mesh to give intended meaning (31 ). Since each of the utterances used in the experimental trials (such as “The banana is to the left of the apple”) was in itself very simple, both grammatically and semantically, we did not suppose that the potential differences between the gifted and average children in the number of regressions might be occasioned by the differences in their verbal abilities. Instead, we presumed that in the context of our research design, the number of between-word regressions should directly bear on the level of metacognitive monitoring and the use of metacognitive strategies, in a similar vein as reported by Kinnunen and Vauras (35, 36). In other words, we presume that the children who by means of their metacognitive monitoring detect that they still do not fully succeed in building an appropriate mental model(s) and consequently are uncertain with regard to the task question will re-read all or only some utterances more attentively and more intently, which should manifest in the number of regressions. Similarly to the fixation duration, the number of regressions was registered within each AOI, the numbers from all AOIs in one trial were added up and the resulting figures for all trials taken by one participant were summed up to yield a single score.

**Gaze transition entropy**. Apart from the differences in time devoted to completing individual trials and from the length of fixations and number of regressions, the variance in metacognitive strategies should manifest also in the overall pattern of gaze movements across the stimulus field. For instance, the children with highly developed metacognitive skills may initially read all four utterances and the question, subsequently inspect the mental model they have built up and if they are still uncertain about some of its parts, they would deliberately focus on the specific utterance that gives the information needed to fill the gap, and this process may be repeated several times in case of need. This routine results in an ordered and highly structured sequence of AOIs visited during the processing of the trial. Conversely, children who are unable or unwilling to apply any reasonable strategy may re-read the utterances in a purely haphazard fashion, which leads to a predominantly random sequence of visited AOIs. The metric that captures whether the scanpath across AOIs is directed or randomly distributed is called gaze transition entropy (sometimes also transition matrix entropy, 31).

Gaze transition entropy (GTE) is computed according to a formula:

(1) $$\text{H }\;\left(R\right)=-\sum_{r_{i}\in R}^{}p\left(r_{i}\right)\log_{2}p(r_{i})$$

where **R** is a normalized transition matrix and r_i_ are the cell values of that matrix with probabilities p(r_i_). The normalized transition matrix has an equal number of rows and columns as the number of AOIs. The value of the cell in C^th^ row and B^th^ column mark the proportion of gaze transitions that started in the AOI C and finished in AOI B, out of all transitions that occurred during the presentation of the trial. Repeated fixations within the same AOI (i.e. the diagonal elements of the normalized transition matrix) do not enter into the computation. The formula (1) gives the value of entropy in bits. If we divide this figure by the maximal theoretical entropy that given combination of AOIs may produce, we get the so called normalized entropy. Its value ranges from 0 to 1. The value of 1 marks maximal entropy, i.e. the gaze pattern is fully random and consequently, all transitions between individual AOIs are equally probable. The value of 0 indicates maximum regularity, this extreme is computationally attainable only when the whole stimulus field comprise a single AOI. The values of GTE were computed independently for each trial, thus, in the subsequent analyses each participant was represented by a vector of 8 elements.

### Procedure

All research sessions took place in the schools of the participants. The first phase (i.e. the administration of the intelligence test) was carried out by way of group testing in ordinary classrooms, with no more than 20 students present at a time. Students were instructed according to the guidelines provided by the test manual. Apart from the examiner a regular teacher of the students was present to ensure that the children who finished early should not disturb their classmates who were still working.

An eye-tracking session took place on a different day, in a separate, quiet study room provided by the school. As the research was carried out in three different schools, three different rooms were used (in each school, the recording always took place at the same place). All rooms were chosen so that they had windows facing northward or westward, all sessions took place in the morning or closely to noon. During each experiment, the blinds in the windows were pulled down and the room was illuminated by standard ceiling white fluorescent lamp or lamp with white LED bulb.

**Figure 3.**
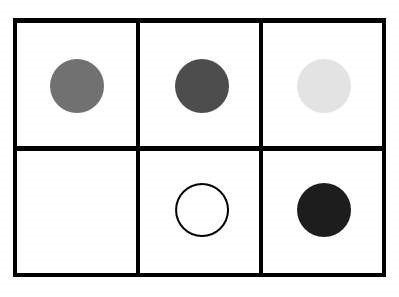
The layout presented on the first training task, in which a following set of premises was used: 1. The green token is to the left of the red token. 2. The red token is to the left of the yellow token. 3. The white token is in front of the red token. 4. The blue token is in front of yellow token. What is the relation between the white and the blue token? Actually, the colored tokens were used, on this illustration the colors are rendered in grayscale.

At the beginning, the participants were presented five training items, aimed at getting them acquainted with the tasks and explaining to them how to proceed. Three of these training items were administered without the computer and with the aid of concrete, physical objects that could be manipulated by the examiner, other two training items were presented on the computer screen in the same fashion as the real experimental trials. None of these training trials were recorded or evaluated. On the presentation of the first training item, a paper with a printed grid (see Figure 3) was laid in front of the child. The examiner then read four utterances describing the position of five tokens (green, red, yellow, white and blue) and at the same time placed these tokens into the corresponding cells of the grid. Then he read the final question asking about the relation between the white and the blue token. The examiner then let the child answer, and pointed out that the response to the question could be worked out despite the fact that this relation was directly described in none of the preceding utterances. The examiner also remarked that a similar task can be solved without seeing the objects in their physical form, just through imagining the layout in one’s mind. The second training task was very similar, except that the structure of utterances was congruent with two distinct mental models. On the presentation of the third training task, the examiner shielded the grid while placing concrete objects (key, coin, dice, rubber and peg) on it and only after the response of the child, he repeated the response, removed the screen and, pointing to the layout, provided a feedback (whether the answer of the child was right or wrong).

After this initial instruction, the participants moved in front of the computer screen with an attached eye-tracking device and they went through another two training trials (reading the assignment themselves this time). After each of these trials, they were also asked to rate its difficulty on a 10-point scale.

Before the presentation of the 8 experimental trials, each participant was calibrated on 5 points. Calibration was considered successful, if the maximum average deviation of the participant did not exceed 0.5°. Participants were instructed to deal with each trial as long as they needed, no time limit was set for the whole procedure. When the participants believed they had arrived at the right answer, they pressed an arrow key to proceed to an empty screen, which appeared after each presentation slide. Only then did they give a verbal answer and the difficulty rating, which was noted down by the examiner. By clicking the arrow key, the participants proceeded to the next trial. On the experimental trials, the participants no longer obtained the feedback regarding their accuracy. The whole procedure, including the initial instruction and presentation of the training items, typically took up about 15-20 minutes for each child.

## Results

Before testing the hypotheses themselves, we ran two preliminary analyses pertaining to the overall success rate in the experimental trials and to the duration of the trials. As for the success rate, we presumed that the gifted children would solve more of the items correctly. Since this assumption is trifling in itself, we did not articulate it as a stand-alone hypothesis, nevertheless we checked it at the beginning of the data processing, because if it did not hold, it would cast serious doubts on the construct validity of the whole experimental design. Leven’s test for the equality of variances indicated acceptable homogeneity of variance between the group of gifted children and the group of average children, *F* (1, 52) = 0.106, *p* = 0.747, thus allowing to use t-test for the comparison of both groups. A one-tailed, independent-samples t-test confirmed that the gifted children scored significantly better, *t* (52) = 2.137, *p* = 0.037, |*d*| = 0.581. In the case of the overall duration (time devoted by the participants to complete the trials) we sought to establish whether there exists a difference between gifted and average children, without assuming beforehand the direction of this difference on the grounds that both directions are theoretically justifiable. On the one hand, gifted children might be expected, by virtue of their higher cognitive abilities, to deal with the tasks more effectively and hence more quickly in comparison to their average peers. On the other hand, a greater propensity for metacognitive monitoring anticipated in gifted children might lead them to check the correctness of each partial step in their problem solving more thoroughly, thus extending the overall time devoted to one trial. For this reason, a two-tailed independent-samples t-test was used. The Leven’s test indicated adequate homogeneity of variance, *F* (1, 52) = 1.229, *p* = 0.273, the results of the t-test showed that the gifted children were significantly faster (*M* = 410788 ms) than their average peers (*M* = 624233 ms), *t* (52) = - 5.525, *p* < 0.001, |*d*| = 1.504. On testing the hypothesis H2, this information needs to be taken into account to forestall finding spurious differences brought about by the time factor.

**Figure 4.**
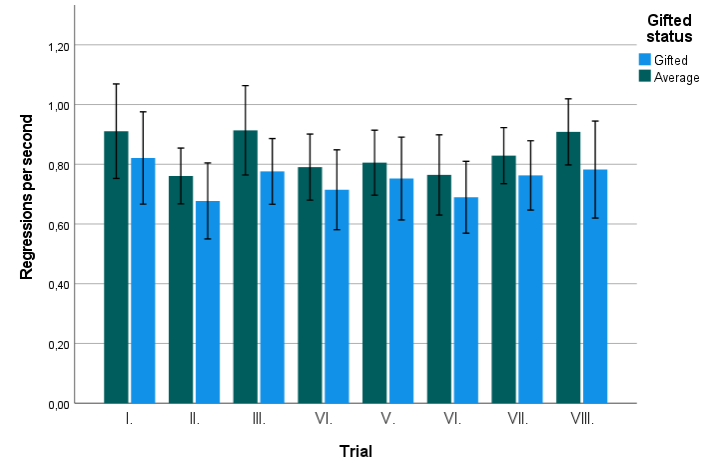
Mean fixation durations in gifted and average children across individual trials. Error bars mark 95% CI.

To test hypothesis H1, the difference between gifted and average children in the fixation duration was analyzed by means of non-parametric Mann-Whitney U-test. This analysis revealed that the difference in the mean fixation duration between both groups was not significant, *z* = -1.028, *p* = 0.304. Figure 4 depicts the size of mean fixation durations, separately in gifted and average children across individual trials.

**Figure 5.**
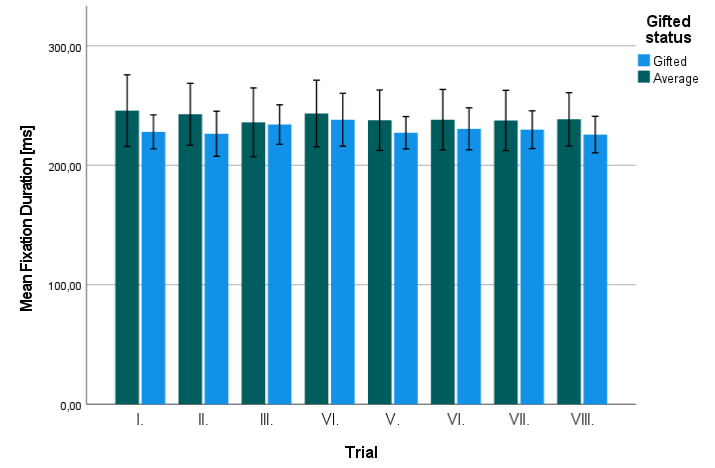
Regression rate in gifted and average children across individual trials. Error bars mark 95% CI.

On testing H2, we had to deal with the fact that the overall number of regressions is naturally highly dependent on the overall trial duration and, as we have seen, both groups significantly differed in this respect. To eliminate this confounding factor, an ancillary variable “number of regressions per second” (regression rate) was created for each participant through dividing the overall number of regressions in the experiment by the overall trial duration. The differences between gifted and average children in this variable were tested by means of the non-parametric Mann-Whitney U-test. This analysis revealed that the differences in the number of regressions per second between both groups were not significant either, *z* = - 1.206, *p* = 0.228. The size of regression rate, separately in gifted and average children across all trials, is shown in Figure 5.

To test the hypothesis H3, an independent-samples Hotelling’s T^2^ test was performed. We used the permutation version of the test (13; 16), which does not make the assumption of multivariate normality of the data. The procedure consisted in comparing two matrices of the type n x p, m x p respectively, where *n* represents the number of participants in the gifted group, *m* represents the number of participants in the average group, and *p* represents the number of individual experimental trials (which was 8 for all participants). Thus, for instance, the item on the third row and second column of the first matrix represented the value of normalized gaze transition entropy of the 3^rd^ participant in the gifted group, measured on the presentation of the second trial. The number of permutations was set to B = 10 000. The computation was carried out without using the shrinkage option for the underlying covariance matrix.

The overall test statistics was *F* = (8, 22) = 0.421, *p* = 0.897. Thus, Hotelling’s T^2^ test failed to detect significant difference between gifted and average children in their levels of normalized gaze transition entropy across experimental trials.

To test the hypothesis H4, we first computed ordinary Pearson correlations between the overall EoSJ ratings and overall trial duration for each group (gifted and average children) separately. In case of gifted children, the correlation was *r_gift_* = 0.496. The value of this correlation coefficient significantly differed from zero, *t* (25) = 2.858, *p* = 0.008. In the group of average children, the correlation between EoSJ ratings and trial duration was *r_avrg_* = -0.022. The value of this correlation, predictably, was not statistically significantly different from zero, *t* (25) = -0.112, *p* = 0.911. In the next step, we tested the statistical significance of the difference between correlation coefficients for the gifted and average group, by means of procedure outlined by Glass and Hopkins (22). First, we carried out the Fisher Z-transformation for both correlation coefficients. This transformation yields a statistic designated Z (to preclude confusion with common z-score, we – along with Glass and Hopkins – always mark this statistic with capital Z). Since the Z statistics has approximately normal distribution regardless of the value of the correlation in the base population, it allows using simple z-test to examine the difference between two sample correlations. The Z statistics is computed as the hyperbolic tangent of the sample’s Pearson correlation coefficient, Z = tanh^-1^r. We then computed the classical z – statistic according to the formula:

(2) $$z=\;\frac{Z_{1}-\;Z_{2}}{\sigma_{Z_{1}-Z_{2}}}$$

where Z_1_ is the value for the sample of gifted children and Z_2_ is the value for the sample of average children.

The standard error of the difference that figures in the denominator, is computed according to the formula:

(3) $$\sigma_{Z_{1}-Z_{2}}=\sqrt{\frac{1}{n_{1}-3}+\frac{1}{n_{2}-\;3}\;}$$

where n_1_ is the number of participants in the sample of gifted children and n_2_ is the number of participants in the sample of average children.

**Figure 6.**
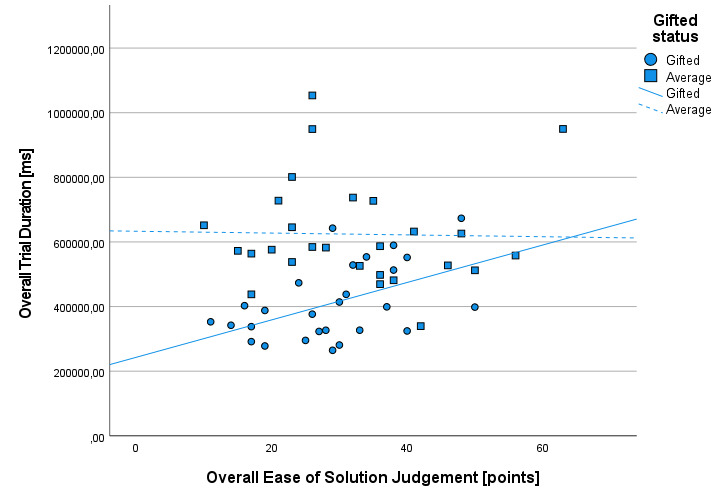
The scatterplot depicting relation between overall EoSJ and overall trial duration, with inserted lines showing linear trend, separately for gifted and average children.

The difference of the correlation coefficients between both groups is statistically significant, *z* = 1.96, *p* = 0.05. Thus, gifted children who perceived the cognitive tasks as more difficult devoted more time for dealing with them, while such relation does not manifest itself in case of their average peers. The scatterplot showing the relation between EoSJ and overall trial duration is presented in Figure 6.

## Discussion

The aim of this study was to investigate into possible differences between gifted and average children in their metacognitive strategies and in their applying metacognitive monitoring. These differences were supposed to manifest themselves in several metrics obtained by means of recording eye movements of the participants. The research has given mixed results: only one of our hypotheses – in our opinion the most important one, though – was confirmed, the remaining three hypotheses do not seem to hold true. In the concluding part of this paper, we try to provide some interpretations of these findings and to discuss the possible implications for future research.

We consider the most important result of the study to be the corroboration of our assumption (as stated in the hypothesis H4) that gifted children are more capable to adapt their problem-solving strategies to the concurrent results of their metacognitive monitoring. Specifically, the gifted students in our sample tended to devote more time to the tasks they perceived as more difficult, whereas in average students this relation seemed virtually non-existent. Owing to the overall small size of the sample, the difference of correlation coefficient between both groups was just at the border of statistical significance on alpha = 0.05 (although the significance of the correlation itself in the gifted group, i.e. its difference from zero, was more considerable). It is also apposite to point out that the value of the effect is at best only moderate. However, some previous work of other authors, carried out along similar lines, lends further support for the conclusion that gifted children are generally more proficient than their average peers in the domain of metacognitive monitoring and in their ability to reflect the results of that monitoring in their problem-solving behavior. For instance, Andrzejewska and Stolińska (7) realized a survey with students in lower secondary education, in which the students were presented with various tasks pertaining to several school subjects (e.g. mathematics, biology, physics etc.), while their eye-movements were registered, and they also provided subjective ratings of the tasks’ difficulty. The study compared two groups of students that differed as to the level of their cognitive abilities, even though the authors did not use the term “gifted” and they chose the highly able group on the grounds of the previous success of these students in a physics competition, without administering a standardized intelligence test to them. The authors then analyzed the relation between subjective difficulty ratings and various eye-tracking metrics, including the temporal ones (overall trial duration and total fixation duration). Although the researchers tested only the significance of individual correlations (i.e. the significance of their difference from zero) and did not perform the significance tests of their differences between groups, the correlations were higher for all examined metrics in the highly-able group compared to average students. These results and their interpretation concur with our conclusion that highly able children are more effective in making use of the outcomes of their metacognitive monitoring.

At the same time, we failed to detect significant differences between gifted and average children in the normalized gaze transition entropy, in the mean fixation duration and – when controlling for overall trial duration – in the number of inter-word regressions. These negative findings may have many explanations, we will present here three of them, the ones that we consider most important and most likely.

In the first place, the explanation may be predominantly developmental. It is natural that metacognition, like other cognitive faculties, is subject to some developmental pattern. Preschoolers and children at the beginning of their school attendance show only rudimentary signs of metacognitive abilities, and they acquire these abilities only gradually in the course of their primary and secondary education. As was convincingly demonstrated by Veenman and Spaans (76) and by van der Stel and Veenman (69), the acquiring of metacognitive abilities in the years of school attendance follows certain specific developmental trajectories. At first, children develop and begin to use certain metacognitive strategies that are tightly bound up with specific cognitive tasks. Thus, a particular young child may already be able to use metacognition in doing mathematical computation, but at the same age he or she is not able to use metacognition at all in learning from a written text, and vice versa. Alternatively, some children of young age may be able to act metacognitively in different cognitive tasks, but use different set of strategies in solving each of them. It is only at the beginning of adolescence that originally isolated metacognitive skills tend to coalesce into a single, broad ability, which the individual is capable to use across a wide variety of learning contexts. One may argue – and with reason – that in gifted children the whole process may be accelerated. Indeed, in the literature on giftedness, the concept of precocity is often emphasized (11), taking as the defining trait of gifted children the fact that they show some cognitive abilities at an extraordinary young age. However, it is plausible to suppose that even in gifted children there is some lower age limit, before which certain metacognitive strategies cannot develop. This problem can be demonstrated in the following example: Bicknell and Levy (10) derived theoretically that certain reading strategy, namely the one in which participants make relatively short first pass fixations and at the same time exhibit an increased number of inter-word regressions, should lead to better learning outcomes in terms of comprehension accuracy. Such strategy can be easily grasped by means of eye-tracking and Weiss (80) reported a study which lends some empirical support to this model. However, this study was realized with adult participants (pre-graduate students) and thus it provides no information whether the same model holds for children and if not, what age represents the watershed after which one’s cognitive system works predominantly in an adult-like fashion. It is thus theoretically possible that in the primary grades there is actually no difference between gifted and average children in the strategies that can be captured by means of eye-tracking metrics we opted for, but if we had used the same experimental design with older students, the effect would have shown up. Consequently, in some future eye-tracking study on the relation between metacognitive and intellectual abilities, it might be beneficial to use the same research design across several age cohorts, ranging at least from primary school children to undergraduate students, and analyze the profile of basic metrics (such as fixation duration or number of inter-word regressions) in each cohort. To our knowledge, no such study has been realized yet.

In the second place, the negative findings might result from the particularities of our stimulus tasks. For instance, in the extant eye-tracking literature, the regression count – in healthy subjects without cognitive impairments – is typically considered as a sign of more attentive reading or as an adaptation to a more complex and more demanding text to be read (60; 80). However, the most influential models which attempt to explain the nature of inter-word regressions construe this increased difficulty primarily in terms of more complex syntactical-semantic structures in the text (for example, more regressions are likely to occur when reading sentences with many relative clauses, containing words with ambiguous meaning etc.), which is not the case of our experimental stimuli. The sentences that our participants read were – in order to forestall any potential effects of differing verbal abilities – deliberately constructed in a very simple vein: as short, syntactically unambiguous, consisting of high frequency, familiar words which denote concrete and easily imaginable objects. The real difficulty of the tasks stemmed from the necessity to retain a relatively high amount of information in working memory while this information was being processed. Before running the experiment, we assumed implicitly that the participants would frequently re-read the utterances while building the corresponding mental model. However, on closer look, a different procedure of the participants might seem equally plausible: they might have read the utterances, once or several times, and then refresh their content in the working memory by silently repeating them, until the target mental model was complete. During such procedure, they might have only rarely needed to check some part of the information by looking at the text again. During such silent rehearsal, the eyes would be supposed to rove indiscriminately across the text, which would explain the lack of difference both in fixation duration and in the number of regressions, as well as the almost identical values of the gaze transition entropy. As for the gaze transition entropy itself, it should be also noted that this metric has come into extensive use only recently, and is still applied quite rarely. In a comprehensive study made by Shiferaw et al. (64), the authors cite only less than 30 papers which report analyses using this metric. Moreover, the vast majority of this research worked with pictorial materials used as stimuli (such as pictures, photographs or visual simulations of some specific environment), rather than with textual ones. Consequently, the theoretical interpretations of GTE developed so far are relevant primarily for explaining its values (and possible differences of them) on viewing visual scenes. Dependable theoretical framework for explaining the GTE on working with textual stimuli has yet to be built.

The third possible way how to interpret our negative findings regarding the hypotheses H1 through H3 may consist in supposing that gifted children adapt their problem solving strategies to the results of the concurrent metacognitive monitoring predominantly (or even exclusively) by means of temporal adjustments. This means that when these children encounter a task which seems distinctly difficult to them they simply devote more time to processing the task (for instance by re-reading the utterance several times, by prolonged thinking etc.), but they do not modify their reading style in the way predicted by Bicknell and Levy (10) nor do any similar “low-level” adjustments. This third explanation is partly supported by the results of another study of ours (52), in which we compared the process of learning from ambiguous texts in gifted adolescents with high and low level of metacognitive skills. Although both groups differed in the time spent in specific AOIs, the differences as regards the number of inter-word regressions were negligible after controlling the effect of time.

Last but not least, it is necessary to stress out again one great limitation of our study, which consists in a relatively small sample size. This is problematic especially as regards the hypothesis H4 since it was confirmed on the very border of statistical significance, and the significance was set at a rather liberal .05 level at that. Despite some other supporting evidence mentioned above, it is realistic that the observed relation might be disproved by some future research carried out on a larger scale. In a similar vein, a prospective future replication might arrive at markedly different effect sizes with regard to the measures of fixation duration, the number of regressions and GTE. The interpretation of our negative findings pertaining to these measures is all the more complicated by the paucity of similar research, to which they could be compared. For instance, we have knowledge of no study using the GTE for the sake of surveying metacognition, much less metacognition in gifted children. It is also apposite to mention that the strength of the study might be improved not only by increasing the number of *participants*, but also by expanding the number of *trials*. It would certainly be worthwhile to administer up to several dozens of deduction problems with more types of underlying mental model arrangements. However, based on our own experience with the experiment, we are certain that such undertaking would be impossible to complete within a single, or even within two or three individual sessions.

To conclude, in our study we provided a modest contribution to the growing notion that gifted children generally differ from their average peers not only in the metacognitive knowledge, but also in the domain of metacognitive skills. We believe that the topic is still important and deserves further study. It is, however, a matter of consideration, whether the eye-tracking represents an effective and purposeful tool for this endeavor. If gifted children adapt their problem-solving strategies solely by means of devoting more or less time to specific tasks or to certain parts of the tasks, then registering eye-movements data will bring only little additional value. It is, of course, true that temporal data are reflected in some of the widely used metrics (such as total dwell time). Nonetheless, if the time is the only variable that counts, other methods of determining it might prove more feasible and more economical. On the other hand, our negative findings may also be perceived as an incentive to use different metrics in the future eye-tracking research on metacognition. It has been reported that alternative metrics such as the number of blinks or pupil dilation relate significantly to the cognitive processing (7 ; 31; 70). Measuring these data is generally more complicated in comparison with the measurement of metrics such as fixation count or fixation duration, because they are more sensitive to external factors such as the level of illumination, and should thus be registered in standardized laboratory settings. This was the reason why we did not make use of these metrics in our study and it is also the reason why they are less employed in eye-tracking research in general. However, if the kinds of data that are more easily acquired and analyzed turn out not to reflect actual differences in the metacognitive skills between different groups, the use of alternative metrics may prove the only way to employ the eye-tracking technology in the research on metacognition in the gifted.

### Ethics and Conflict of Interest

The authors declare that the contents of the article are in agreement with the ethics described in http://biblio.unibe.ch/portale/elibrary/BOP/jemr/ethics.html and that there is no conflict of interest regarding the publication of this paper.

### Data availability

The dataset for this study is available at Figshare repository (www.figshare.com). DOI: 10.6084/m9.figshare.14233391

### Acknowledgements

This study is a result of the research funded by the Czech Science Foundation as project GA17-14715S "Development of Metacognitive Skills in Gifted Children”.

The paper was written with the support of the Specific University Research (project no.: MUNI/A/1458/2020 – “Domain Specific Abilities of Gifted Students”) provided by the Ministry of Education, Youth and Sports of the Czech Republic.

We would like to thank HUME Lab - Experimental Humanities Laboratory of Masaryk University for lending us the instrumental equipment necessary for the realization of the study.
